# A Novel Fluoride Containing Bioactive Glass Paste is Capable of Re-Mineralizing Early Caries Lesions

**DOI:** 10.3390/ma11091636

**Published:** 2018-09-06

**Authors:** Ahmed Samir Bakry, Mona Aly Abbassy, Hanin Fahad Alharkan, Sara Basuhail, Khalil Al-Ghamdi, Robert Hill

**Affiliations:** 1 Operative Dentistry Department, Faculty of Dentistry, King Abdulaziz University, Jeddah 21589, Saudi Arabia; 2 Conservative Dentistry Department, Faculty of Dentistry, Alexandria University, Alexandria 21614, Egypt; 3 Department of Orthodontics, Faculty of Dentistry, King Abdulaziz University, Jeddah 21589, Saudi Arabia; monaabbassy@gmail.com (M.A.A.); haneenharkan@gmail.com (H.F.A.); sara.basuhail@hotmail.com (S.B.); 4 Dental Department, Alexandria University, Alexandria 21614, Egypt; 5 Primary Health care Centre, Sabt Alalayah Hospital, Ministry of Health, Bisha 67611, Saudi Arabia; khlilsaeed@gmail.com; 6 Physical Sciences in Relation to Dentistry, Institute of Dentistry, Dental Physical Sciences Unit, Queen Mary University of London, London E1 2AD, UK; r.hill@qmul.ac.uk

**Keywords:** fluoride bioactive glass, biomin, caries, enamel

## Abstract

White-spot-lesions (WSL) are a common complication associated with orthodontic treatment. In the current study, the remineralization efficacy of a BiominF^®^ paste was compared to the efficacy of a fluoride gel. Methods: Orthodontic brackets were bonded to 60 human premolars buccal surfaces, which were covered with varnish, except a small treatment area (3 mm^2^). All specimens were challenged by a demineralization solution for 4 days. Specimens were assigned into 4 groups: BiominF^®^ paste, Fluoride (4-min application), fluoride (twenty four hours application), and the control (*n* = 15). After cross-sectioning, enamel slabs having a thickness of approximately 100–120 μm were obtained. A TMR (Transverse Micro Radiography) technique was used to observe the sub-surface enamel lesions’ depth and mineral density, and their response to the remineralization protocols. One way ANOVA was used to analyze the results (α = 0.05). The top and the cross-sectional surfaces were observed using SEM/EDS. Results: Specimens treated with BiominF^®^ paste showed significant decrease in delta z values, however lesion depth showed no significant difference when compared to the other three groups (*p* < 0.05). SEM/EDS observation showed the formation of crystal like structures on top of enamel demineralized surfaces, when treated with BiominF^®^ paste. In conclusion BiominF^®^ paste can be considered an effective remineralizing agent for white spot Lesions.

## 1. Introduction

Many orthodontic patients seek improvement in their dental aesthetic features; however orthodontic treatment is often associated with the development of white spot lesions, which is extremely difficult to treat [1]. Moreover, progress of these lesions to form cavities occurs frequently in many cases, thus jeopardizing the final expected esthetic results [1]. The increase in caries risk factors in orthodontic patients may be attributed to the presence of many retentive areas surrounding the enamel brackets, which retain large quantities of cariogenic oral biofilm [2]. Previous studies showed that the levels of cariogenic biofilm in the oral cavities of orthodontic patients, may be 2–3 times higher than in normal individuals suffering from high rates of biofilm formation [3].

Moreover, the spread of dietary habits and assorted systemic [4,5,6] and genetic disorders [7], may lead to the development of various early enamel lesions, which need re-mineralizing [8] rather than surgically treating these lesions through composite restorations. The need for development of efficient remineralizing agents, encouraged researchers to investigate the possibility of using bioactive glasses, for the re-mineralization of enamel [9,10] and dentin lesions [11,12].

Results reporting the efficacy of using bioactive glasses in treating dental lesions, have suggested that adding small amounts of calcium fluoride to a bioactive glass may enhance the formation of fluoroapatite, which is expected to exhibit acid resistance to acidic cariogenic attacks [13].

In the current study, the trans-microradiography technique was used together with the SEM/EDS technique [9,10] to investigate the capability of the BiominF^®^ paste as a potent remineralizing agent.

The hypothesis adopted in the current study was that the BiominF^®^ paste will be able to re-mineralize the artificial white spot lesion induced on the enamel surfaces.

## 2. Materials and Methods

### 2.1. Specimens’ Preparation

**Figure 1:**
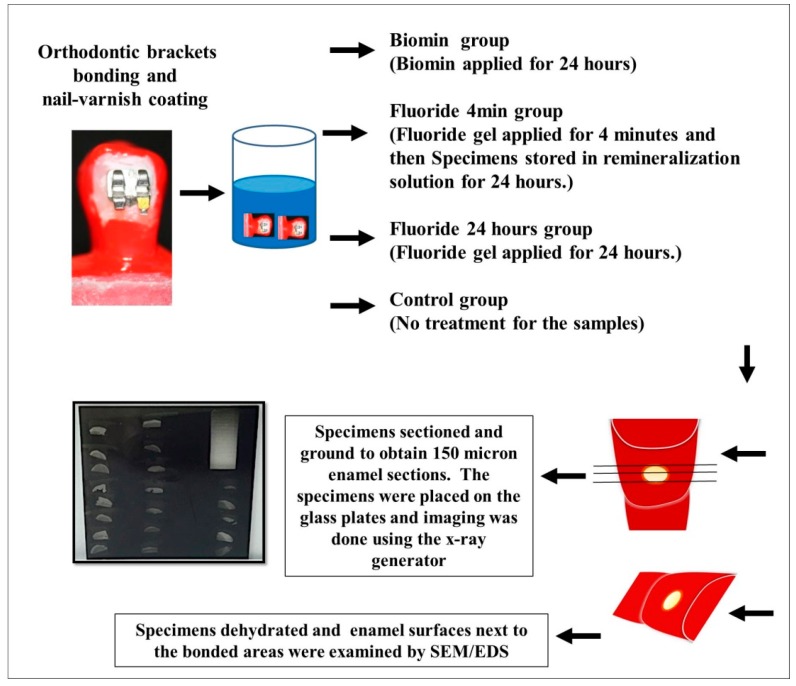
Samples preparation and examination.

The teeth’s buccal surfaces were cleaned thoroughly using pumice. Unitek™ Etching Gel (3M Unitek, Monrovia, CA, USA) was applied exclusively to the enamel surfaces, onto which the orthodontic brackets were bonded for 15 s, followed by rinsing using distilled water and drying by air-way syringe for 15 s. The orthodontic metallic brackets (MiniSprint^®^, Forestadent, Pforzheim, Germany) were carefully bonded to the buccal surfaces of all specimens, without extruding any of the Transbond XT primer or the Transbond PLUS color change adhesive (3M Unitek) to the area of observation, next to the cemented Orthodontic brackets [1]. All specimens were embedded in resin material. Preparation and examination of the specimens are summarized in (Figure 1).

The nail-varnished specimens were stirred in the demineralization solution (2.2 mM CaCl_2_, 10 mM NaH_2_PO_4_, 50 mM acetic acid, 100 mM NaCl, 1 ppm NaF, 0.02% NaN_3_; pH 4.5), for 4 days using a low-speed (100 rpm) magnetic stirrer [10]. The demineralized specimens were assigned into 4 groups (*n* = 25). There were 25 specimens that had a fluoride gel (1.23% acidulated-phosphate-fluoride, Gelato Gel, NJ, USA) (fluoride group) applied for 4 min and then gently wiped by a moist gauze; 25 specimens had the same fluoride gel applied and was not washed away (Fluoride-24 h), but rather covered by a protective light-cured resin material layer for 24 h; 25 specimens had BioMinF^®^ applied on its surface (Biomin group); while the rest of the specimens received no further treatment (control group). All specimens were stored in a remineralizing solution (1.0 mM CaCl_2_, 3.0 mMKH_2_PO_4_, 100 mM acetate, 100 mM NaCl, 0.02%, NaN_3_; pH 6.3) according to Reference [6], for 24 h [10].

### 2.2. Biomin Application

One tenth of a gram of BioMinF^®^ powder composed of (22–24 mol % Na_2_O, 28–30 mol % CaO, 4–6 mol % P_2_O_5_, 36–40 mol % SiO_2_, and 1.5–3.0 mol % CaF_2_) was mixed on a glass slab with 2 drops of 50 wt% phosphoric acid, which was prepared by the diluting 85 wt% phosphoric acid (Wako, Osaka, Japan). The resulting paste had a pH 2.5. The BioMinF^®^ paste was applied onto the enamel surfaces of the (Biomin group) by microbrush. The aforementioned method of application was utilized previously, for the application of another bioactive material [1,9,10,11,12,14].

### 2.3. Application of Bonding Agent

All specimens in the BioMinF^®^ or Fluoride-24 h groups had their remineralizing agents protected by a layer of bonding agent (Clearfil SE Bond, Kuraray-Medical, Tokyo, Japan), which was applied over these remineralizing agents then light-cured [1,9,10,11,12,15,16,17,18] using LED (Woodpecker™ LED Curing light, China with an output of 900 mW/cm^2^). After storage in the re-mineralizing solution for 24 h, the temporary bonding agent layer was removed carefully by a sharp instrument, as was previously described in References [1,9,10,11,12,15,16,17,18].

### 2.4. TMR Analysis

Fifteen specimens from each group were assigned for the TMR analysis. Preparation of the specimens started by dehydrating them in ascending alcohol solutions, followed by immersing the specimens in styrene monomer, and then finally embedding the specimens in low-viscosity polyester resin (Rigolac, Oken, Tokyo, Japan) in a special mold. The aforementioned low-viscosity resin, has the ability to penetrate the porous enamel surface resulting from the demineralization process. The resin penetration into the enamel surface would help in reinforcing the demineralized enamel surface and decrease the incidence of sample cracking to be 10% of the total utilized samples. After the polymerization of the embedding resin, the specimens were cut using a low-speed diamond saw (Isomet; Buehler, IL, USA) and were ground by SiC abrasive papers having different grits (ranging from 800 till 1200 grit) to obtain sections, which were approximately 100 to 120 μm in thickness [14,19,20,21]. The x-ray generator (CMR 2; Softex, Tokyo, Japan) was adjusted to generate 20 kV voltage and 2 mA currents for 10 min. All specimens were placed on a sensitive X-ray glass plate film (High Precision Photo-Plate-PXHW, Konica, Tokyo, Japan). The TMR images, together with 15 aluminum step-wedges, were captured in the sensitive glass plate film, and were digitized using a digital camera attached to a microscope (ML 8500, Meiji, Techno, Japan). The relative mineral density (%) was calculated as was previously reported in References [14,19,20,21]. The definitions of lesion depth and the mineral loss (∆*Z*, vol % μm), followed the previously published data in Reference [14,19,20,21].

### 2.5. SEM/EDS Top Surface Examination

The white spot lesions that were formed in five specimens from each group, were examined by SEM/EDS (JCM-6000 NeoScope, JEOL, Tokyo, Japan). All specimens were dehydrated followed by gold coating. The specimens’ surface and chemical composition were observed by SEM/EDS (JCM-6000 NeoScope, JEOL), for the following elements phosphorus, calcium, oxygen, fluoride, carbon, oxygen, and silicon.

### 2.6. SEM/EDS Interface Preparation

Five specimens from each group were embedded in light cured resin system, according to the manufacturer’s instructions. The specimens were then cross-sectioned perpendicular to the interface to give 2-mm-thick slabs. The transverse sectioned surfaces were ground and then polished with diamond pastes, down to 0.25 µm. The specimens were dehydrated then gold coated [13]. The cross-sections of the interface were examined using the SEM/EDS (JCM-6000 NeoScope, JEOL), according to Reference [22]. Line scans were done using the EDS attachment, across the treated enamel surfaces to detect the following elements: phosphorus, calcium, oxygen, fluoride, carbon, oxygen, and silicon.

### 2.7. Statistical Analysis

The results of the mineral loss (∆Z, vol % μm) and lesions depths of all groups were analyzed using one-way ANOVA, followed by a Tukey test (*p* < 0.05). (The software used was SPSS 10.0 (SPSS Inc., Chicago, IL, USA)).

## 3. Results

### 3.1. Transverse Microradiography

**Figure 2:**
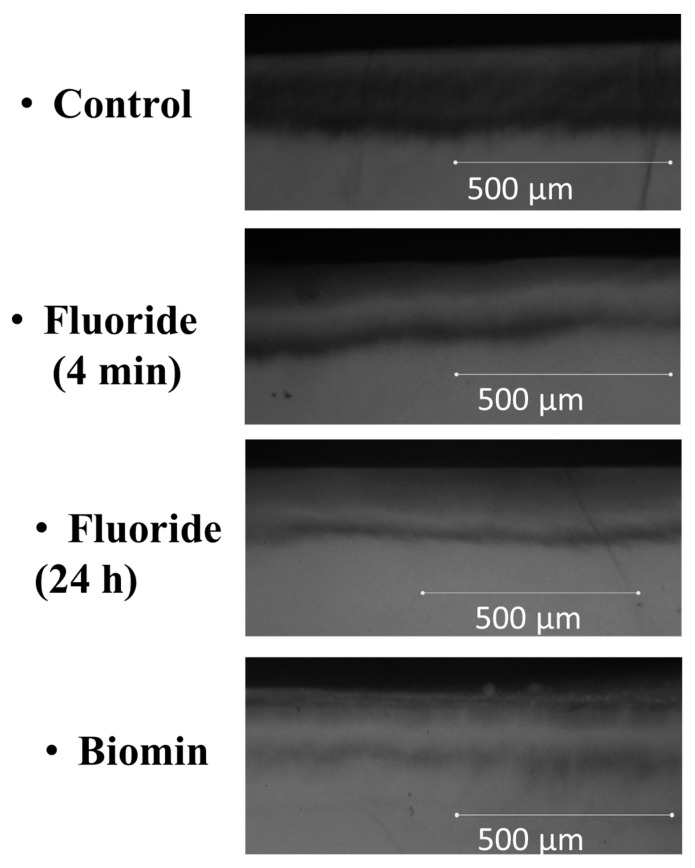
Transverse Micro Radiography (TMR) images for the four groups.

**Figure 3:**
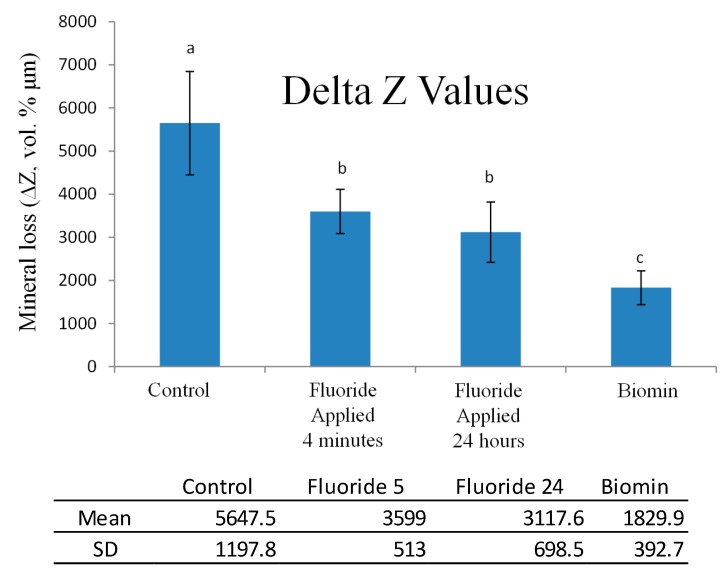
Mineral loss (ΔZ) after treatment protocols presented by bar chart and Table. Bars with same letters were not statistically significant α = 0.05.

**Figure 4:**
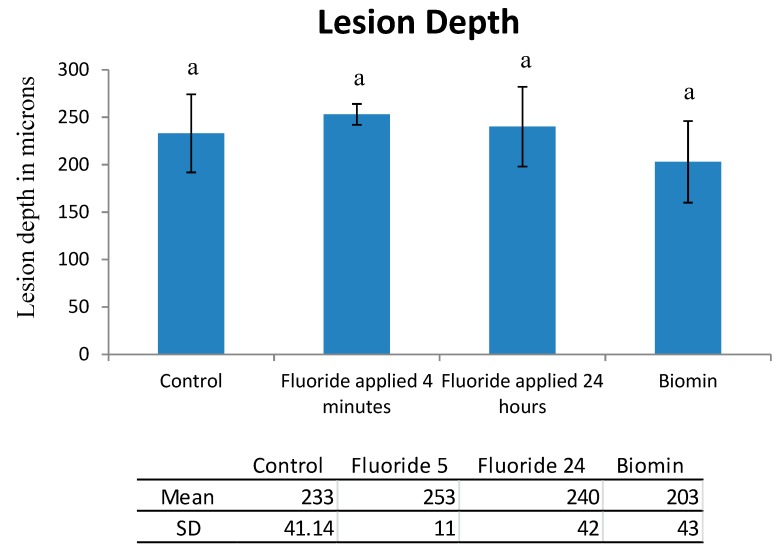
The results of Lesion Depth in µm, presented by bar chart and Table, showing significant decrease in lesion depth for the Biomin group, compared to the remaining groups. Α = 0.05. Bars with same letters were not statistically significant α = 0.05.

The TMR images obtained for specimens of the 4 groups, are shown in Figure 2. The means and standard deviations of ∆Z, and lesion depth, are shown in Figure 3 and Figure 4. The Biomin group showed statistically significant reductions in ∆Z values, but there was no significant reduction in lesion depth values for the Biomin group, when compared to the rest of specimens in the remaining groups (*p* < 0.05).

### 3.2. SEM/EDS Top Surface Examination

**Figure 5:**
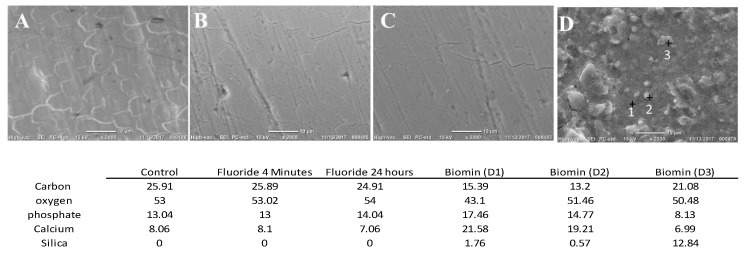
Top surface examination by SEM/EDS for the four groups. (Control) group (**A**), the (fluoride 4 min) (**B**), and the (Fluoride-24 h) (**C**) show the boundaries of the enamel prisms. Biomin group (**D**) shows the formation of a newly formed layer having crystal like structures and silica particles. The table shows the mass percentages of each element detected by EDS in each group.

The (Control) group, the (fluoride-4 min), and the (Fluoride-24 h) groups’ examination showed signs of enamel demineralization, with obvious boundaries of the enamel prisms (Figure 5A–C); indicating the weak remineralizing potential of the fluoride containing agents with different application modes, utilized in the current study. EDS results showed decreased values for the mass% of the phosphorus and calcium. On the other hand, the Biomin group showed the deposition of crystal like structures, covering the whole de-mineralized enamel surface (Figure 5D). EDS analysis showed that the aforementioned layer was rich in calcium and phosphate contents, with trace amounts of silica.

### 3.3. SEM/EDS Interface Examination

**Figure 6:**
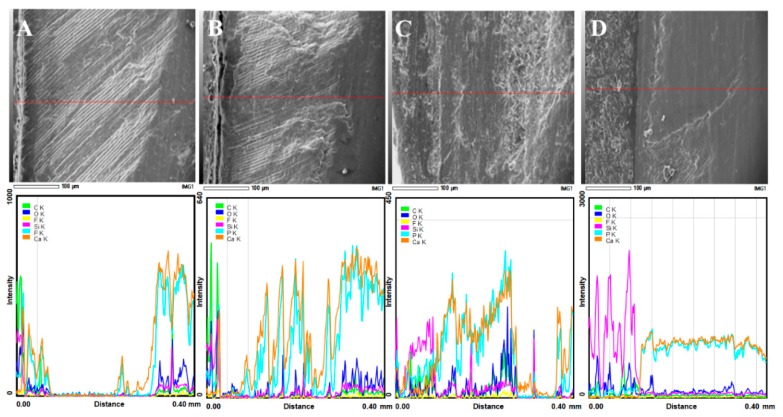
The (Control) group (**A**), the (fluoride 4 min) (**B**), and the (Fluoride-24 h) (**C**) specimens showed demineralization of the subsurface area, with deterioration of the micro-morphological features of the demineralized areas. Biomin group (**D**) showed remineralization of the subsurface areas, with strong calcium and phosphate peaks in the surface re-mineralized areas.

Specimens of the control group, the fluoride 4 min group, and the 24 h fluoride showed a decrease in the calcium and phosphorus peaks, as line scans crossed from the enamel to the embedding resin material (Figure 6A–C). The outer most enamel surface showed some weak peaks of calcium and phosphate however; these peaks were extremely weak when compared with the peaks of calcium and phosphate of normal enamel. Moreover, the subsurface enamel lesion topographical features showed roughness of the surface, indicating the presence of multiple voids and defects created between the enamel crystals in the demineralized enamel lesion.

**Figure 7:**
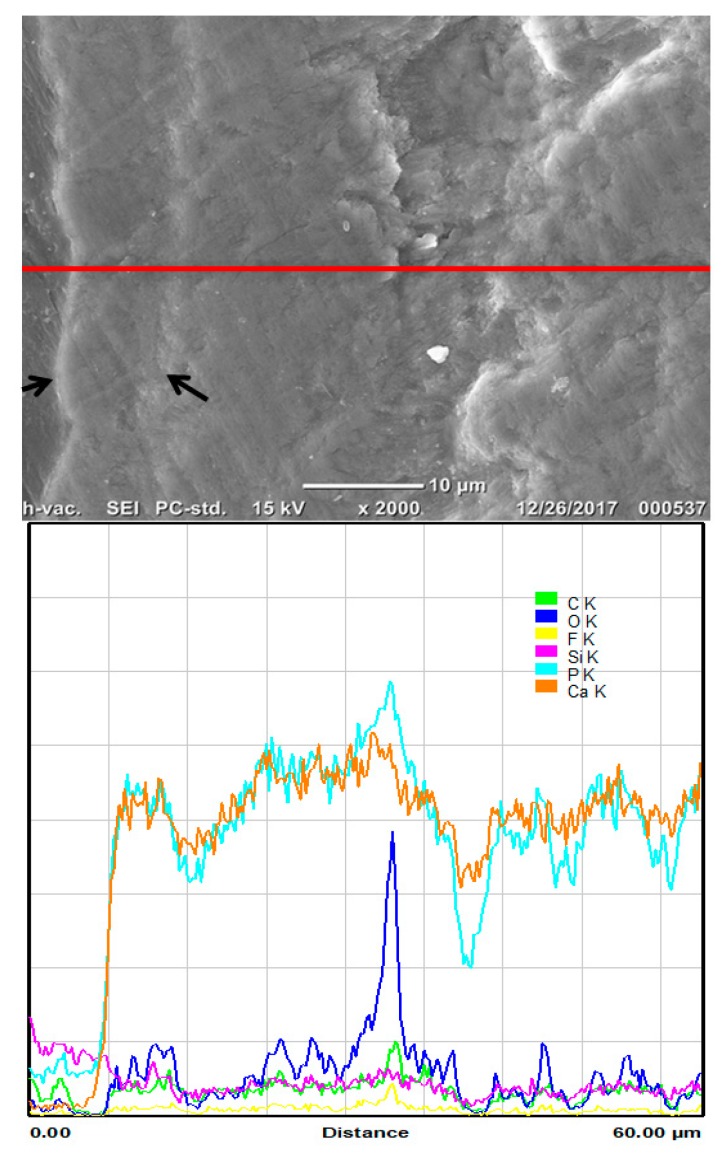
Examination of the Biomin group (D) with high magnification. Pointers are pointing to the newly formed layer on the demineralized enamel with strong calcium and phosphate peaks.

Specimens of Biomin group (Figure 6D and Figure 7) showed the formation of a calcium phosphate rich layer on top of the enamel surface, and the subsurface enamel area exhibited a smooth area.

## 4. Discussion

The null hypothesis adopted in the current study was accepted. Previous research [22], showed the difficulty of re-mineralizing the sub surface demineralized enamel lesions, due to the difficulty of localizing significant high concentration of calcium, phosphate, and fluoride ions to promote the effective enamel subsurface remineralization. However, in the current experiment, biomin was capable of remineralizing the sub-surface enamel lesion efficiently, as was demonstrated by the results of the TMR experiment. TMR study is considered the gold standard for conducting experiments for testing the remineralization capacity of any agent, as described in References [19,20,22,23].

To test the remineralization efficacy of Biomin to treat the sub-surface enamel lesion, we compared its efficacy to the application of an agent containing a high concentration of fluoride (9000 ppm fluoride). Previous research [24,25], showed that agents containing high concentration of fluoride are associated with better remineralization to enamel lesions. The approximate lesion depth of the demineralized subsurface lesion induced in the current study exceeded 200 microns, previous studies showed that subsurface enamel lesions of about 100 microns needed approximately 28 days to re-mineralize in artificial saliva, without being exposed to any demineralization challenges [26]. This may explain the poor remineralization capacity of the fluoride agents utilized in the current study, which was observed by TMR. The poor fluoride remineralization pattern detected in the current study may be attributed to; firstly, the short application period, i.e., 24 h or 4 min; and secondly, the high affinity of the fluoride ions to form fluoroapatite when combined with hydroxyapatite crystals present in the superficial layers of the demineralized enamel. This might have decreased the penetration of the fluoride ions into the deeper layers of the enamel sub-surface lesion [9,23].

On the other hand, the low fluoride content of the BioMinF^®^ (in contrast to the high fluoride content of the utilized fluoride gel), allowed the penetration of BioMinF^®^ rich content of calcium and phosphate through the porous enamel sub-surface, causing the re-mineralization of the demineralized enamel lesion rather than remineralizing the outer enamel surface, as was observed when using fluoride as a sole remineralizing agent. Moreover, previous research showed that the presence of low concentration of fluoride in the BioMinF^®^ glass facilitated apatite formation, since fluorapatite forms at about a unit of pH lower than hydroxyapatite, and fluoride is known to catalyze the conversion of brushite, octacalcium phosphate, and amorphous calcium phosphate to apatite [27]; which may explain the decrease in the roughness of the BioMinF^®^ glass specimens’ SEM-EDS images, due to the repair action exerted by the BioMinF^®^ paste [13,27,28,29].

Based on the obtained results from the TMR and the SEM/EDS examination, it is suggested that the mechanism of forming the apatite layer after the BioMinF^®^ may be summarized as follows: the BioMinF^®^ powder that was mixed with 50% phosphoric acid released calcium, phosphate, fluoride, and sodium ions onto the demineralized enamel surface [9,20,21], causing the mobilization of some calcium and phosphate ions from the enamel surface [1,5,6,7,8,23]. The phosphate ions released from BioMinF^®^ powder and the diluted phosphoric acid solution, in addition to the calcium ions released from BioMinF^®^ were preserved from being diluted in the storage media (Saliva in case of clinical application) by the action of the protective bonding agent layer. The high concentration of calcium and phosphate ions will tend to move to the deep areas of the sub-surface enamel lesion, rendering this sub-surface lesion saturated with calcium and phosphate ions capable of repairing the voids and defects resulting from the acidic challenge. Moreover, some calcium and phosphate ions will be catalyzed by the presence of the fluoride ions to form a layer of acidic calcium-phosphate salts, on top of the demineralized enamel surface (as was observed by the SEM/EDS examination) [9,19,20]. The detection of trace amounts of silica on top of the enamel surface after Biomin paste application, may be attributed to the breaking down of the silica network of the BioMinF^®^ by the action of the water content of the aqueous part of the acidic solution used to mix the BioMinF^®^ powder; it was previously reported that the silica network will form the highly soluble Si-OH groups (Silanol groups) [9,20,21], upon being mixed with water, and thus it was expected that most of these silanol containing compounds would be washed out upon being rinsed with water spray after 24 h. The difficulty in detecting the fluoride peaks may be attributed to the low photon energy of the fluoride, which may render it difficult to be detected by SEM-EDS.

The addition of a rich source of phosphate (supplied from the phosphoric acid aqueous solution) to the BioMinF powder, enhanced the formation of a significant layer of a calcium phosphate compound on top of the demineralized enamel, as was previously suggested in Reference [20]. The temporary coverage of the BioMinF paste, by a thin layer of bonding agent for 24 h, protected the calcium and the phosphate ions released by the Biomin paste from being washed out by the storage media, and provided a large reservoir of calcium–phosphate ions to be released into the demineralized enamel lesion [5,6,8,23,30].

The aforementioned hypothesis for the BiominF paste action, was based on the observation of the current study and previous research. It is suggested that further research is needed to confirm the formation of fluoroapatite, and to detect the exact role of fluoride ion concentration in the BiominF paste.

## 5. Conclusions

The application of the BioMinF^®^ paste using the current technique, formed a layer rich in calcium and phosphate on top of the enamel surface, suggesting that the aforementioned layer provided the source of calcium and phosphate ions which re-mineralized the subsurface enamel surface. Storing the samples for 24 h in intimate contact with fluoride 9000 ppm, did not cause significant re-mineralization of the tested sub-surface enamel lesion. The physical properties of the white BioMinF^®^ paste on demineralized enamel using the current technique may be acceptable to patients, because it resembles the application of temporary filling materials. However, clinical studies are needed to confirm this hypothesis and to confirm the clinical remineralization capacity of BioMinF^®^ paste.
